# Virtual Reality for Neuroarchitecture: Cue Reactivity in Built Spaces

**DOI:** 10.3389/fpsyg.2017.00185

**Published:** 2017-02-13

**Authors:** Cristiano Chiamulera, Elisa Ferrandi, Giulia Benvegnù, Stefano Ferraro, Francesco Tommasi, Bogdan Maris, Thomas Zandonai, Sandra Bosi

**Affiliations:** ^1^Neuropsychopharmacology Lab, Sezione Farmacologia, Università di VeronaVerona, Italy; ^2^Altair Robotics Laboratory, Department of Computer Science, Università di VeronaVerona, Italy; ^3^Experimental Psychology Department, Faculty of Psychology, Mind, Brain and Behaviour Research Center, University of GranadaGranada, Spain; ^4^Italian League against Cancer (LILT)Reggio Emilia, Italy

**Keywords:** cue reactivity, setting, context, drug addiction, interior design, architecture, neuroarchitecture, virtual reality

## Introduction

Domestic and urban environments are associated to our life experiences and behaviors. These environments may acquire an emotional and motivational value and, in turn, shape our behaviors. Although there is a well-established knowledge of the effects of built space features on perception, feelings, and affective responses (Ulrich, 1991), only a limited attention has been however paid to physical space-induced motivated behaviors. There is still a strong attitude to consider the control of motivated behaviors as a matter of individual desires, free will, moral choices, executive control, etc.—and not as the interaction between environment and personality, genetics, and brain mechanisms.

Recently, there has been a convergent agreement from architects, designers, psychologists, and neuroscientists about the multifactorial nature of the reciprocal interaction between humans and built space, and how it could impact on well-being psychological distress and risky behaviors (Sternberg, 2009). The emerging interdisciplinary field of “neuroarchitecture” developed conceptual paradigms and empirical frameworks based on the interaction between brain and built spaces (see Academy of Neuroscience for Architecture; www.anfarch.org). Within this framework, we would like to propose the “Cue Reactivity” phenomenon as a paradigmatic example of such as interaction. Cue reactivity (C-R) is the adaptive response to salient information in the environment (Niaura et al., 1988). Salient information is that associated to drugs, sex, palatable food, and to a variety of natural and non-natural rewards (such as gambling, shopping, etc.). Drug C-R manifests itself as an array of responses to stimuli previously associated to drug effect. The detrimental consequence of C-R is relapse to drug-seeking and drug-taking (Rohsenow et al., 1991). On the other hand, C-R is an evolutionary phenotype of the interaction with the environment: in fact, spatial context rich of reward-related cues may stimulate both positive and risky motivated behaviors.

In this Opinion paper, we will show that identification and design of specific physical space features may affect mental health, and that indoor and furniture of drinking venues are associated to alcohol use. Based on what we know about C-R, and on the effects of built spaces on psychological and behavioral processes, we think that more research is now possible to plan and design research-based “C-R-free situations.” For instance, investigations on outdoor and indoor features associated to C-R may help to develop “motivational safer built environments.” The complexity of real world investigations is not however easily modeled in the laboratory, but technologies like virtual reality may offer the possibility to increase subject's presence in a spatial context simulation and, in the meantime, the control of the experimental parameters (García-Rodríguez et al., 2012). For these reasons, we propose virtual reality as a methodological approach in-between naturalistic and experimental lab setting for a better understanding of built space features affecting C-R.

## Cue reactivity and addictive behaviors: the smoking case

Drug C-R response can be measured as changes of desire/wanting, heart rate and skin conductivity (physiological measures), and gestures/actions (behavioral measures) (Chiamulera, 2005). Studies in laboratory animal have shown that molecular and cellular changes correlate with the drug cue effect (See, 2005). Imaging studies in humans showed the activation of brain areas involved in motivational, emotional and cognitive processes (Yalachkov et al., 2012). Yalachkov et al. (2012) also proposed affordance as the process underlying smoking-related action representations in response to C-R. Affordance is the neural representation and the related behavioral outcome of emergent feature from the relationship of an actor, objects, and environment (Tucker and Ellis, 1998). Costantini et al. (2010) showed that the affordance relation is based not only on mutual appropriateness of object's features and on individual's motor abilities, but also on their spatial relationship conditions. Indeed, a prospective study by Gilpin et al. (2006), showed that a smoking home suppresses the efficacy of pharmacotherapy for smoking cessation when compared to a smoking-free domestic environment. Therefore, it appears that not only the discrete stimuli (such as objects) play a determinant role to C-R, but also the living space (the spatial context) is a strong determinant factor for C-R, and subsequent relapse.

## The *conditioned space*: the role of context in cue reactivity

Research in laboratory animals has extensively investigated the conditions under which rewarding drugs confer conditioned properties to the environment, which in turn affects addictive behaviors (Crombag et al., 2008). Badiani et al. showed how either familiarity or novelty of a context might affect acquisition, maintenance, and relapse to drug use in laboratory animals and in addicts (Badiani et al., 2011) suggesting a cross-interference between brain, behavior, and setting. Several studies have been also done in smokers, and the effects of therapeutic interventions have been investigated (Warthen and Tiffany, 2009; Dunbar et al., 2010; Shiffman et al., 2013).

Dewey in “Quantitative Thoughts” (Dewey, 1931) defined as *Pervasive Unifying Quality* the internally integrative nature of experience as time/space units that renders unique the quality of experience. It is from the context of a situation that (perceptually) later emerge objects, people, events that attract attention and that acquire emotional, motivational, and cognitive values. According to Dewey, objects, people, and situations acquire a *meaning* for what they represented in the experience but only if including the *unifying* sense of the contextual situation. In human laboratory studies, Conklin and colleagues systematically investigated the role of environmental context in craving for smoking (Conklin and Tiffany, 2002) based on the assumption that proximal (the discrete cues) and distal (the contextual setting) stimuli are two different categories of variables (Conklin, 2006). Proximal stimuli are discretely defined in terms of structure and properties (Conklin et al., 2008), for instance, a burning cigarette. Distal stimuli are defined as a complex of stimuli that own a conditioned value as a whole, for instance a bar or a social space. Although the real experience of an individual includes proximal and distal stimuli, laboratory research separately investigated features and values of these two categories (Conklin et al., 2010). Differently from proximal stimuli, a complex set of distal stimuli own greater individual specificity. C-R may be induced by either general or personal proximal stimuli within a complex set of distal stimuli; if the latter are from a personal context may induce a stronger C-R. Several studies using Ecological Momentary Assessment (EMA; Shiffman, 2009) investigated the situations associated to C-R for smoking (Ferguson and Shiffman, 2011). The interactions between stimuli (proximal and/or distal) and presence of other people and of allowed/prohibited smoking conditions appear to play a relevant role in the management of cigarette craving. Specific comparisons have been made between home/private vs. public spaces. Although public spaces are characterized by increasing prohibitions, bar and restaurant spaces showed the strongest association to C-R (Dunbar et al., 2010). It therefore appears that research was able to give an empirical demonstration of John Dewey's intuitions about the relevance of the “sense of the situation,” and of its interaction with cues, context and conditions on C-R.

## Evidence-based interior design and architecture

Studies such as the above-cited “EMA” studies and those like Gilpin et al. (2006) suggested the need to consider the importance of outdoor and indoor features as determinant factors to smoking C-R. The effects of space features on affective processes and well-being has been studied especially in the context of healthcare environments (e.g., Cusack et al., 2010; Lankston et al., 2010). Urlich proposed a conceptual framework for evidence-based design of healthcare (Ulrich et al., 2008, 2010) that included general and specific recommendations for built space features affecting mental state and behavior of patients and professional staff. Dolan et al. (2016) developed the “SALIENT checklist” for evidence-supported design based on variables such as “sound, air, light, image, ergonomics, and tint” of built environments (Dolan et al., 2016). Type of doors and walling material, access pathways width and other characteristics of built external environment correlate with different mental health disorders, including alcohol abuse (Ochodo et al., 2014). Built environments rich of conditioned proximal and distal stimuli may therefore induce different adaptive responses (Bradford and Dolan, 2010), similarly to the development of C-R as a form of learning involving neuroadaptive mechanisms (Chiamulera, 2005).

Some studies described urban features that are associated to substance use. For instance, Linas et al. (2015) investigated use patterns for cocaine and heroin in built spaces such as home, church, abandoned space, store, bar, etc. Alcohol use and alcohol-related problems are closely associated to specific physical spaces such as drinking venues (Green and Plant, 2007). The literature on the association between drinking and drinking venues showed that venue style (e.g., shabby décor, low-cost furniture, no theme, etc.) is associated to alcohol use and intoxication (Hughes et al., 2011). The control strategy focused on changing substance use “micro-environments” (Hollands et al., 2013) has recently recommended more research, for instance on alcohol glass shape (Attwood et al., 2012; Troy et al., 2015) as well as on the physical features of the built space such as materials, interior design, and furniture, external wall features, etc.

## The neuropsychology of immersive contextual simulation

**Figure 1:**
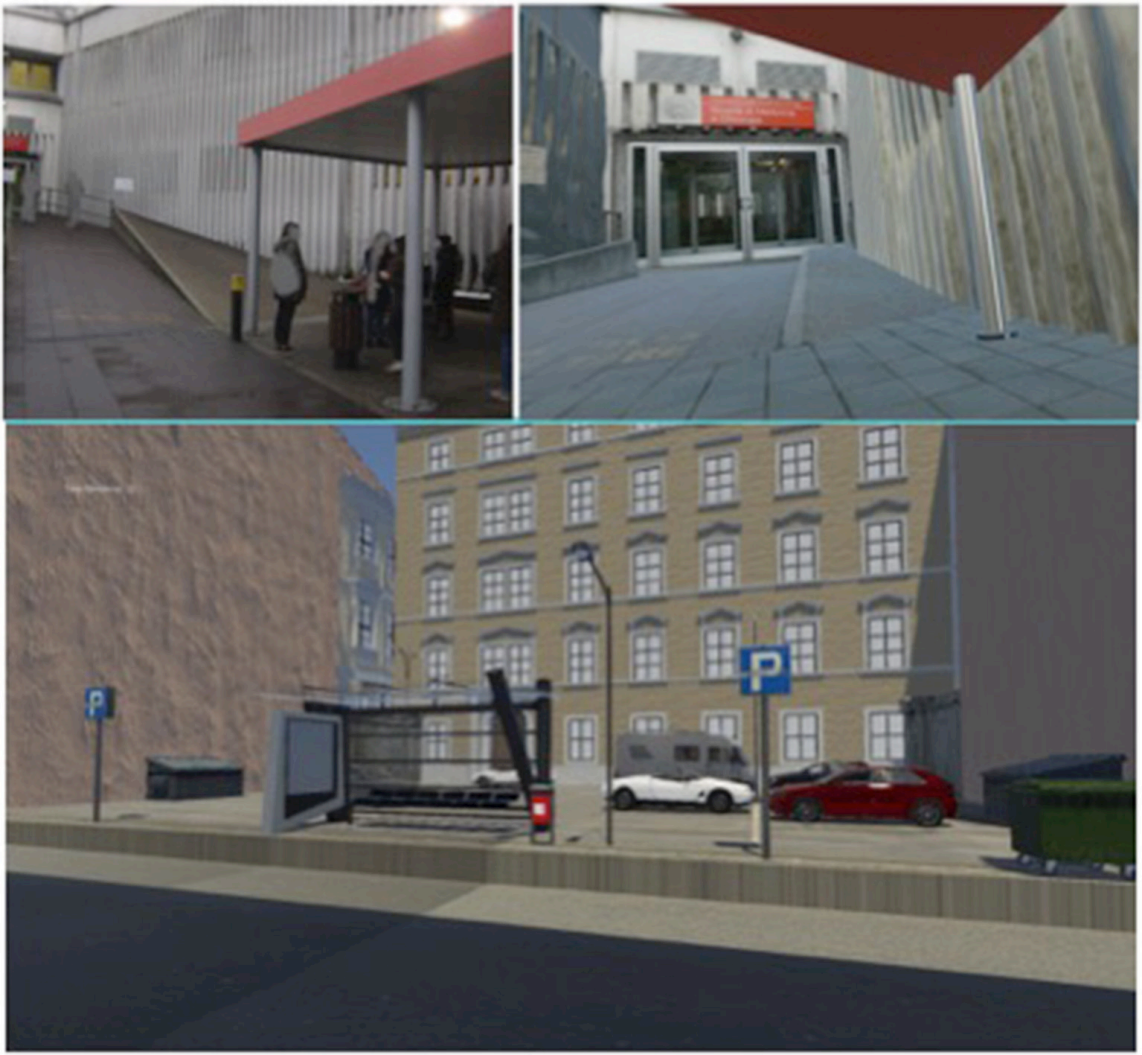
**Examples of outdoor and indoor cue reactivity context**. Real world smoking context (**upper left**: digital photograph) and corresponding virtual reality simulation (**upper right:** Unity 5 simulation screenshot). Virtual simulation of a bus stop (**lower panel:** Unity 5 simulation screenshot).

The difficulty to mimic the real C-R situations in the lab (requiring at the same time controlled complexity and personalization) needs ecologically oriented models (Chiamulera et al., 2007) at a level of analysis between the real situation and the lab setting. In the last few years, the use of virtual reality technologies showed strong validity for different maladaptive behavior, including smoking (Hone-Blanchet et al., 2014). The virtual reality simulation creates a state of “immersion” that comes close to the real situation, allowing the controlled measure of psycho-physiological and behavioral responses (Pericot-Valverde et al., 2015). Virtual reality has been proposed not only as a valid research tool but also as a safe therapeutic intervention (Valmaggia et al., 2016). Besides these advantages, virtual reality may provide a vast array of outdoor and indoor simulation scenarios with variation in color, material, decoration, furniture, room/building type. More specifically for the purpose of C-R research, virtual simulations of personal settings associated to smoking (Pericot-Valverde et al., 2014), as well as to food or drinking, may be easily developed and validated. Our group is currently investigating the effects of immersion in a personalized smoking context rich of general smoking objects, in order to explore the combined effect of personalized distal and general proximal stimuli (Figure 1).

On the other hand, a complementary approach is to identify “motivational positive” contextual features in order to stimulate safer behaviors and healthy life styles. Vecchiato et al. investigated the neuropsychological basis of the interaction with aesthetical features. They showed that appreciation of virtual architecture environments activates electroencephalographic correlates of visuomotor exploration and judgment of pleasure and familiarity (Vecchiato et al., 2015a). They found that these experiences correlated with embodiment (i.e., action possibility into the environment) and motivational processes (Jelic et al., 2016). The effects of pleasurable immersion in an indoor simulation (Vecchiato et al., 2015b) was similar to those taking place when smokers are embedded in environments provided with proximal and distal stimuli that indicate a possibility of action (Casartelli and Chiamulera, 2015). Obviously, the safer outcome of a positive aesthetical experience of “cutting-edge” design is different from the deleterious one of smoking C-R.

## Conclusion

People cannot be left alone taking care of the consequences of their risky behaviors, in particular when affected by disorders that develop a maladaptive and associative learning. Although several therapeutic interventions have been developed for C-R inhibition (Courtney et al., 2016), we strongly recommend an earlier research-based approach to the design of human spaces that might also act as effective preventive intervention. Interdisciplinary collaboration is needed among interior designers, architects, city planner with neuroscientists, psychologists, and healthcare professionals. New laboratory models based on virtual reality may help to identify in the real life those proximal and distal stimuli affecting either positive or negative motivated behaviors.

Such super-creative alliance may therefore provide to the individuals and to the society safer homes and urban context. This “prevention design” will then need to be associated to information and education so that, hopefully, everyone will be able in the future to furnish her/his home and to shape personal living space for a better lifestyle.

## Author contributions

SF and BM setup the virtual reality apparatus and developed the scenarios. EF, GB, FT, and TZ provided the literature sources, designed protocols and procedures, performed the test, analyzed the data. CC and SB developed the concept behind the manuscript, and co-contributed to the logistical and financial support. All the authors contributed to the elaboration of the concept to a publishable topic. CC wrote the manuscript and the co-authors edited the final version.

## Funding

The “5per mille 2012” research grant by the Italian Cancer League (Lega Italiana Lotta per i Tumori, LILT) supported the study (PI: CC) and research grant for GB. LILT also supported CC and SF with educational grants.

### Conflict of interest statement

The authors declare that the research was conducted in the absence of any commercial or financial relationships that could be construed as a potential conflict of interest.
